# Supplementary material for: Trauma-focused treatments for depression. A systematic review and meta-analysis
Source: PLoS One. 2021 Jul 22;16(7):e0254778. doi: 10.1371/journal.pone.0254778 (PMC8297785; doi:10.1371/journal.pone.0254778)
Supplement: Attachment — Submitted filename: Plos One reviewers comments june 2021.docx [file pone.0254778.s003.docx]

Response to reviewers

Thank you for your comments, we have taken on board your suggestions and believe that this has improved the quality of this manuscript. We have addressed your specific comments below. In addition, we have added the following references:

Birrer, E., & Michael, T. (2011). Rumination in PTSD as well as in traumatized and non-traumatized depressed patients: A cross-sectional clinical study. *Behavioural and cognitive psychotherapy, 39*(4), 381-397.

Cowdrey, F. A., & Park, R. J. (2012). The role of experiential avoidance, rumination and mindfulness in eating disorders. *Eating behaviors, 13*(2), 100-105.

Dickson, K. S., Ciesla, J. A., & Reilly, L. C. (2012). Rumination, worry, cognitive avoidance, and behavioral avoidance: Examination of temporal effects. *Behavior therapy, 43*(3), 629-640.

Mihailova, S., & Jobson, L. (2018). Association between intrusive negative autobiographical memories and depression: A meta‐analytic investigation. *Clinical psychology & psychotherapy, 25*(4), 509-524.

Newby, J. M., & Moulds, M. L. (2010). Negative intrusive memories in depression: The role of maladaptive appraisals and safety behaviours. *Journal of affective disorders, 126*(1-2), 147-154.

Payne, A., Kralj, A., Young, J., & Meiser-Stedman, R. (2019). The prevalence of intrusive memories in adult depression: A meta-analysis. *Journal of affective disorders, 253*, 193-202.

And removed:

Hofmann, A., Hilgers, A., Lehnung, M., Liebermann, P., Ostacoli, L., Schneider, W., & Hase, M. (2014). Eye Movement Desensitization and Reprocessing as an Adjunctive Treatment of Unipolar Depression: A Controlled Study. *Journal of EMDR Practice and Research, 8*(3), 103-112.

Holmes, E. A., Blackwell, S. E., Heyes, S. B., Renner, F., & Raes, F. (2016). Mental imagery in depression: Phenomenology, potential mechanisms, and treatment implications. *Annu Rev Clin Psychol, 12*.

*Reviewer #1:*

*I appreciate the responses to the original comments. I still have concerns that the current data does not fully support the claim that TFTs are an effective treatment for depression, given that aside from one study only EMDR is examined. Additionally, the way that depression is being discussed does not fully capture the diagnosis or depression symptoms outside of adversity exposure, which does not occur for all individuals who experience depression. I have expanded on these concerns below.*

1. *The types of adversity you are highlighting as leading to depression (e.g., neglect, parental divorce, bullying) are those that are often discussed in research on developmental trauma disorder or complex trauma, proposed diagnoses that are meant to capture exposure to chronic trauma, particularly in childhood (see Pearlman & Courtois, 2005; Van der Kolk et al., 2009). Moreover,* *causes of depression are not always adversity-based, further highlighting a need to soften these claims. They can be biological, related to lack of achievement/purpose, loss of intimacy, feeling out of control. Individuals with a history of adversity may have more severe symptoms, as you mentioned, and these adversities may not lead to a PTSD diagnosis based on the limitations of the PTSD criteria, but again this is not representative of major depression as a diagnosis - this is representative of the experience of a subset of individuals whose symptoms may actually be captured by a complex trauma diagnosis.*

Thank you for your comment. In the paper we cite several sources that identify the well-established link between adversities and depression (e. g. Laugharne, Lillee, & Janca, 2010; Nanni, Uher, & Danese, 2012; Risch et al., 2009). While we are not proposing that this is the only factor that impacts on the likelihood of depression or the severity of diagnosis, we are proposing that, similar to biological factors, it is a significant predisposing and maintaining factor for many who have the disorder, and one that is largely neglected in depression interventions. In patients with a trauma-related disorder the characteristics of the traumatic event itself can be different than in patients with depression. In the first case the event has to be related to actual or threat of death, physical or sexual violence, but in the case of depression these events can have a totally different character. In a previous paper treated people with depression without soliciting whether they had a history of adverse events or not (Dominguez, Drummond, Gouldthorp, Janson, & Lee, 2020). The types of traumas that were treated are detailed in the slide below (Dominguez, 2021). In this study we were not treating those patients who would make criteria for complex trauma. These patients would have needed many more sessions to achieve remission of their depression diagnosis. In our revised submission we have elaborated on the prevalence of intrusive memories, and related avoidance and rumination in depressed individuals and the role of these in symptom exacerbation and maintenance as suggested.

2. *I would like to see some discussion of rumination in the context of your reference to intrusive memories, similar to how you reference avoidance. Rumination is a core feature of depression that often occurs as a strategy to manage intrusive memories and contributes to the frequency/maintenance of intrusive memories. This would support your argument further. Moreover, I would recommend including the review by Payne et al. (2019) to add to the support of the associations between intrusive memories and depression, given that this is more recent than the Wheatley & Hackman (2011) citation and offers some language around why trauma-focused treatments such as EMDR may be beneficial, while also acknowledging limitations.*

Thank you for this comment. We have altered the introduction by updating the information from the Payne et al. (2019) and *Mihailova & Jobson (2018*) paper and also added information regarding rumination.

3. *Though I continue to think the language around TFTs being beneficial for depression should be tempered, I ultimately defer to the editor as I think the findings do make a contribution to the field and highlight the need to further explore TFTs as a treatment for depression. I would, however, like to see the recommendations in the paragraph above addressed, as I think a discussion of rumination in the context of depression/intrusive memories is important (the recent meta-analysis by Mihailova & Jobson, 2018 may be useful) and the updated Payne et al. cite/examination of those findings will add to the paper.*

As stated above we have included material on rumination and the references you provided which strengthened the paper. We have further altered the abstract and discussion to be more circumspect around the evidence for TFT in general.

*Reviewer #2:*

1. *Please do a read-through for typos/grammar (e.g. “defied” instead of “defined).*

Thank you. This has been addressed.

2. *I think it would be helpful to clarify further in the “Selection Criteria” section that participant studies did not require a diagnosis of PTSD (or required NO diagnosis of PTSD?).*

We have added the following sentence into the PICO for clarity: *“Studies that required participants to have a PTSD diagnosis were excluded from our analysis.”*

3. *I agree with the first reviewer that it still reads to me as a bit misleading in the first and last paragraphs of the discussion to state that TFTs are promising (when really we’re just talking about EMDR). While I understand that the authors set out to examine TFTs (so it makes sense to still label the paper and hypotheses in that way), it seems there should be a middle ground in the discussion that better represents what was found.*

We have altered both the abstract and discussion to ensure this is clearer.

4. *It may make sense to mention that if we are examining TFTs for adverse events, that perhaps it no longer makes sense to refer to them as TFT’s (perhaps adversity-orientated treatments?).*

This is an interesting idea and our research group discussed this at length. We come to the decision that trauma therapy are common terms used both in the literature and clinical practice and that we can something that neglect, abuse etc are traumatic and that treatments that target anything that is traumatic can be called trauma focused treatment. In thinking through this issue and what is generally meant by the terms we did come across several clinical discussion sites where the concept of possibly two main categories of trauma commonly referred to as Big “T” and little “t.” Big “T” traumas are the events most commonly [associated with post-traumatic stress disorder](https://journeypureriver.com/ptsd-triggers-substance-abuse/) (PTSD) including serious injury, sexual violence, or life-threatening experiences. Little t traumas are any life events experienced by the individual as traumatic but would not fit the PTSD category A diagnostic criteria. We think that the treatment of each should be the same. We thought about writing this up in the discussion but decided it appeared outside the scope of the paper. However we would do this if the editor felt otherwise.

5. *One differential between PTSD and depression is “intrusive” thoughts vs “ruminative thoughts”. This made me wonder if depression studies have found that people with depression experience thoughts that are truly “intrusive” in the way defined by PTSD.*

We expanded our information on intrusive memories to further specify related avoidance and rumination. We also added a sentence in the discussion to identify that future work in this area is needed.

References

Dominguez, S. (2021). *EMDR for depression.* Paper presented at the EMDR Europe Research & Practice Conference, Virtual.

Dominguez, S., Drummond, P., Gouldthorp, B., Janson, D., & Lee, C. W. (2020). A randomized controlled trial examining the impact of individual trauma‐focused therapy for individuals receiving group treatment for depression. *Psychology and Psychotherapy: Theory, Research and Practice*. doi:10.1111/papt.12268

Laugharne, J., Lillee, A., & Janca, A. (2010). Role of psychological trauma in the cause and treatment of anxiety and depressive disorders. *Current Opinion in Psychiatry, 23*(1), 25-29.

Nanni, V., Uher, R., & Danese, A. (2012). Childhood maltreatment predicts unfavorable course of illness and treatment outcome in depression: a meta-analysis. *American Journal of Psychiatry, 169*(2), 141-151.

Risch, N., Herrell, R., Lehner, T., Liang, K.-Y., Eaves, L., Hoh, J., . . . Merikangas, K. R. (2009). Interaction between the serotonin transporter gene (5-HTTLPR), stressful life events, and risk of depression: a meta-analysis. *Jama, 301*(23), 2462-2471.
